# Drooping genes v/s dancing genes

**DOI:** 10.4103/0019-5545.55080

**Published:** 2009

**Authors:** T. S. Sathyanarayana Rao, K. S. Jagannatha Rao, M. R. Asha

**Affiliations:** Department of Psychiatry, JSS University, JSS Medical College Hospital, M.G. Road, Mysore - 570 004, India; 1 Departments of Biochemistry and Nutrition, Central Food Technological Research Institute, Mysore - 570 020, India; 2 Reiki Practitioner and Freelance Writer, 788/160, 18^th^ Cross, Ramanuja Road, Mysore, India

Exploring avenues for stimulating and harnessing the tremendous self-healing potential of the human genome, is one of the hot spot areas in current research.

A prime candidate for illness is a person whose life is devoid of love or of any level of human warmth. Add to this, unresolved or deeply consuming emotional, psychological or spiritual stress along with a disempowering belief system-you have a perfect recipe for creating disease.

When a person feels that he or she has no choice in the matters of his or her own life, their emotional response can lead to the development of a disease. The violation of a person's choice, often, is teeming with hostility, fear, anger and rage.

‘The Lost Soul Syndrome’, when a person is in ‘Existential Vacuum’, where suffering is accompanied by the absence or loss of meaning in one's life, could be a significant contributor to illness. This is primarily because, a life sans meaning, more often than not, leads to despair, depression and worthlessness. This implies that the physical body is strongly affected when one's state of mind and emotions are full of suffering, stemming from feelings of emptiness.[1]

Life is a learning experience. By redirecting our focus, we bring about a new course of events into focus while releasing an existing course of events that may not serve us any longer. Nowhere this is more significant than where our genes are concerned.

Of late, geneticists have begun to address the DNA (NOT the Brain) as our Bio-computer. It can be hypothesized that, in order to set the “bioenergy system” right, it is vital to access the source of electromagnetic malfunction in our genetic code. To facilitate this, it is essential to employ special sounds and intention.[2]

According to Dr. Valerie Hunt, the author of “Infinite mind: Science of the Human vibrations of consciousness”, the mind actually exists at the dimension of electromagnetic fields, rather than residing in the brain. Natalie Dobrova believes that, an individual's system comprising organs, has its own electromagnetic rhythm. Disruption in this cellular symphony signifies disease.[3]

DNA is regarded as the master blue print for our biology. Vedas state that “In the beginning was Brahman with whom was the WORD”. A parallel for this can be found in The New Testament where John states “In the beginning was the WORD.” A reference in Genesis 1:3 reads “And God *said*, Let there be light: And there was light.” All these point to the potential of light (electromagnetic waves) and sound (electromagnetic waves) on DNA and their EXPRESSION.[2]

Horowitz, the author of “DNA: Pirates of the sacred spiral”, states that the research carried out by neurophysiologists has clearly shown the following: “One-third of the sensory-cortex area of the brain is fully devoted to the oral cavity, tongue, lips and speech.” This implies that bioacoustic waves (oral frequency emissions- spoken or sung) have tremendous impact over life and vibrating genes which in turn influence total wellness of individuals and probably affect evolution of the species too.[2]

Dr. Jeremy Norby, a French anthropologist, conducted detailed studies on Healing techniques of Shamans in the Amazon forests, has also authored the book “The Cosmic serpent: DNA and the Origins of Knowledge”. He compared DNA or genetic code to books, which can be read, written or REWRITTEN (Reprogrammed). Now comes the important piece of the puzzle – the significance of ‘Junk DNA’ – parts of the DNA called “Introns” which are regarded as noncoding genetic sequences, in contrast to “Exons” that have identifiable coding functions in protein building through transcription. It is also interesting to note the presence of “Jumping DNA” or “transposons” in the supposedly useless 97% of junk DNA.[2] It is proposed that these mysterious junk DNA have some important function since mother Nature never does anything dumb.

Horowitz hastens to add that, according to three Nobel laureates in medicine, the primary function of DNA lies not in protein synthesis (less than 3%) but in the reception and transmission of electromagnetic energy (more than 90%) functioning in the realm of bioelectric (light) and bioacoustic (sound) signaling.[2]

A Russian research team of geneticists and linguists have scientifically documented the ability of sound and light to heal DNA. The research carried out by Russian scientists Peter Gariaev and Vladimir Poponin, showed that DNA has a special ability to attract photons causing them to slide along the helical molecule of DNA instead of along a linear pathway. This implies that DNA has the amazing capacity to bend or wrap light around itself! Recent studies by Dr. Rao and his team[4] showed that DNA undergoes helical change from normal B-DNA conformation to Z-DNA in Alzheimer's disease and this alters gene orientation and function. Thus DNA helicity alters depending on brain function and stress.

Iona Miller and Richard Miller, genetic researchers,[2] conclude that it may be possible to activate DNA through conscious linguistic expressions (sounds-words; intentions-thoughts…forms of electromagnetic energy) to modify the human bioenergy fields, which in turn can affect anatomy and physiology of the human body.

Psychologist Ernest Rossi effectively explores the revolutionary implications of newer discoveries in genetics and neuroscience, in the every day life of humans, for health and healing.[5] In his groundbreaking book “The Psychobiology of Gene Expression”, he examines with fresh eyes, the tremendous application potential of consciously motivated behavior, subjective states of mind and our PERCEPTION of choice and free will in regulating gene expression to optimize health.

Neuroscientists Gage and Kemperman[6] opine that a vital component of medical treatment is the regeneration of damaged/dysfunct neural networks. They also believe that conscious regulation of cognitive or environmental stimuli, changes in our physical activities may be part of doctor's prescription in future, besides or substituting drugs.

The emerging field of epigenetics[6] has amassed mind boggling evidence to show that consciously chosen positive beliefs, feelings and spiritual practices influence our health by modulating gene expression. When we consciously intervene to send epigenetic signals to our cells, these signals can reduce stress and positively impact the synthesis of life-enhancing hormones such as DHEA and many more.

We are now beginning to be aware of and appreciate, as a community, the impact of our thoughts and emotions on our genes. It is now being increasingly recognized that our body is sensitive enough to receive a signal from external environment and turn it into a sequence of electromagnetic or chemical commands for our genes. One such breakthrough experimental finding has been that, without interfering with the mouse's DNA, a small nutritional change in the pregnant mouse could have a dramatic impact on the gene expression of off – spring.[6]

Bruce Lipton, author of the best selling book, “The Biology of Belief” showed that, the environment operating through the cell membrane, modulated the behavior and physiology of the cell, switching the genes on and off. He firmly believes that our beliefs affect the expression of our genes.[7] Extending Lipton's work, researchers at the Institute of HeartMath in California revealed that human emotions, desires and intentions can bring about measurable molecular changes in the DNA.

Rewriting our ‘Life Scripts’ may have the power to turn on or turn off the eloquence of our genes which will have a huge impact on the quality of our lives *per se*. By redefining our reality, we can choose to reshape our lives.
